# Unusual presentation of rheumatoid arthritis in a 106‐year‐old woman: A rare case report

**DOI:** 10.1002/ccr3.7120

**Published:** 2023-03-17

**Authors:** Ali ALQazzaz, Faiq I. Gorial, Ahmed Dheyaa Al‐Obaidi, Mustafa Najah Al‐Obaidi, Nabaa Ihsan Awadh, Hashim Talib Hashim

**Affiliations:** ^1^ Department of Medicine University of Babylon Babylon Iraq; ^2^ Rheumatology Unit, Department of Medicine, College of Medicine University of Baghdad Baghdad Iraq; ^3^ College of Medicine University of Baghdad Baghdad Iraq; ^4^ Rheumatology Unit Baghdad Teaching Hospital Baghdad Iraq

**Keywords:** arthritis, autoimmune, elderly, rheumatoid arthritis, rituximab

## Abstract

By reporting this case, we hope to encourage medical professionals to concentrate on diagnosing old patients with unusual presentation of rheumatoid arthritis.

## Graphical abstract

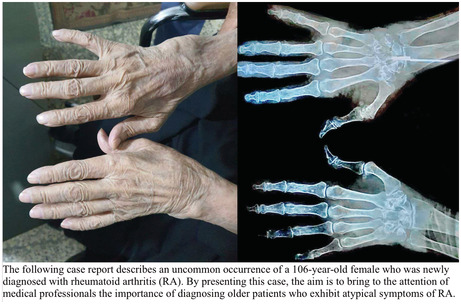

## 1 INTRODUCTION

Rheumatoid arthritis is a systemic autoimmune disease with a peak incidence between the ages of 30 and 50 years old. Rheumatoid arthritis that first appears in people over the age of 60 is commonly referred to as “elderly onset Rheumatoid Arthritis.” We present an extremely rare case of a 106‐year‐old female newly diagnosed with rheumatoid arthritis. By reporting this case, we hope to encourage medical professionals to concentrate on diagnosing old patients with unusual presentation of rheumatoid arthritis.

“Rheumatoid arthritis (RA) is a systemic autoimmune disease primarily affecting synovial tissue and leading to joint destruction and disability”.^1^ Approximately 0.3–1% of the world's population suffers from RA.^2^ RA that first appears in people over the age of 60 is commonly referred to as “elderly onset Rheumatoid Arthritis”.^3^ Traditional estimates place the peak incidence of RA between the ages of 30 and 50; nevertheless, the number of late‐60s onset cases has been rising in recent years.^4^ Elderly‐onset rheumatoid arthritis (EORA) differs from RA that presents before age 60 in a variety of ways, including the prevalence of males, the clinical presentation, the prognosis, the presence of co‐morbid conditions, and the frequency with which an anti‐cyclic citrullinated peptide antibody or rheumatoid factor is positive.^5^, ^6^ We present an extremely rare case of a 106‐year‐old female newly diagnosed with RA.

## 2 CASE PRESENTATION

A 106‐year‐old female presented with generalized bone pain, low‐grade fever, and the inability to walk; she was previously able to walk alone in her home without an assistant. She went to a a general practitioner, who misdiagnosed her with osteomalacia and gave her vitamin D and calcium supplements. Her family history revealed a relative with RA who was treated for 5 years with methotrexate and rituximab.

**FIGURE 1:**
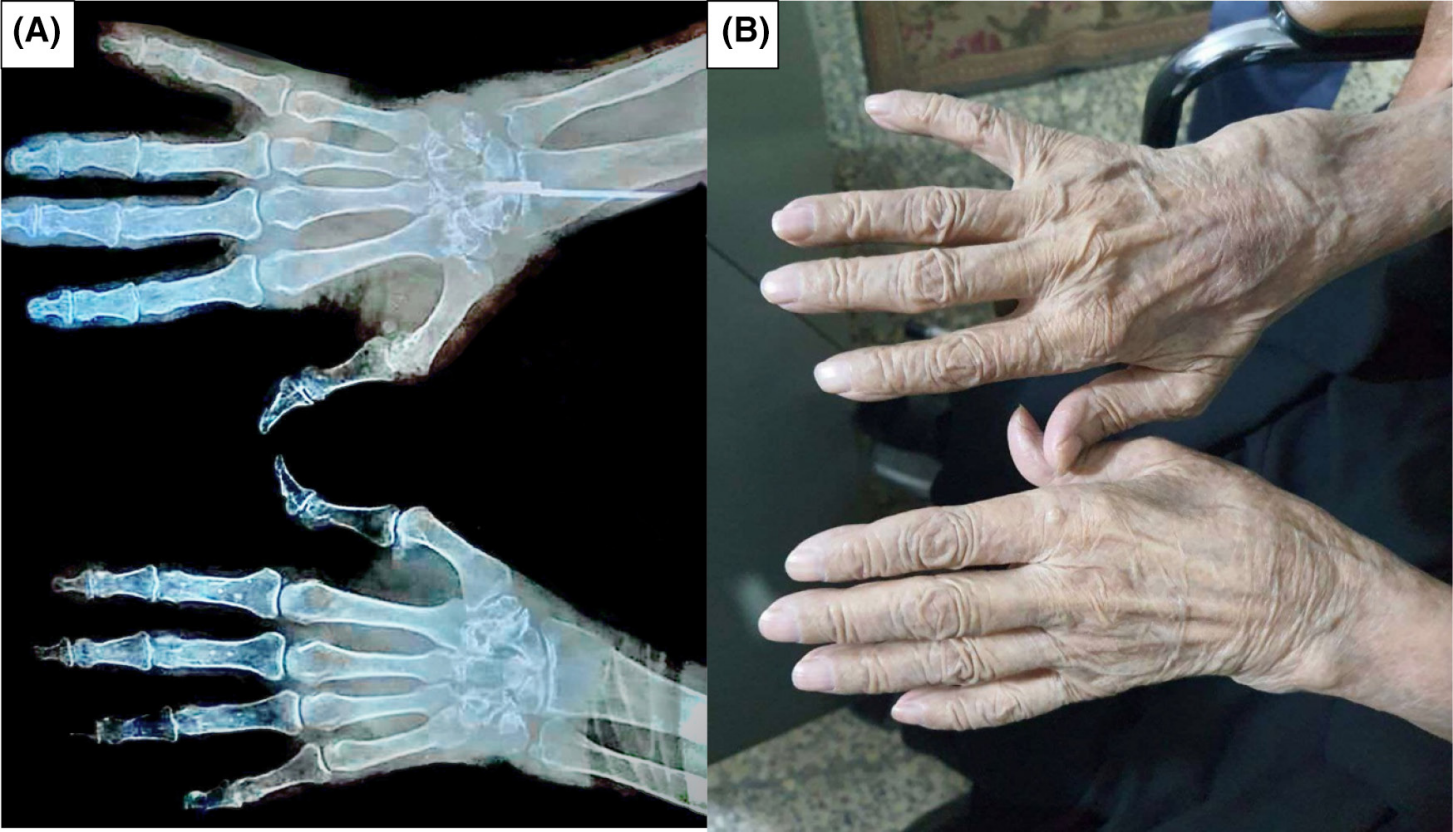
(A) Anteroposterior x‐ray of the hands, showing erosion at the wrists, MCP joints, and PIP joints. (B) Hand deformities: ulnar deviation, *Z*‐shaped deformity of both thumbs and wrists, and MCP joints swelling.

On examination, vital signs were within normal ranges, but she had bilateral wrist swelling, MCP joints swelling, and PIP joints swelling (as shown in Figure 1B) with tenderness, as well as bilateral feet with MTP joints swelling with tenderness. Over the past 3 months, she has been unable to stand on her feet due to pain. She was hypertensive for more than 20 years, and her hypertension is well‐controlled. She is not a smoker. The differential diagnoses were: inflammatory arthritis, crystal arthritis, degenerative arthritis, endocrinopathy causes, and paraneoplastic musculoskeletal symptoms.

Her investigations revealed a hemoglobin of 10 g/dL. The ESR was 65 mm/h, the CRP was positive, and so was the rheumatoid factor. Serum uric acid, liver function tests, renal function tests, and thyroid function tests were all within the normal ranges. The virology screening tests were negative.

The chest X‐ray and abdominal ultrasonography were normal. The hand X‐ray showed bilateral osteopenia with bone erosions (as shown in Figure 1A).

After the diagnosis of RA was confirmed, the rheumatologist prescribed her a low dose of methotrexate (7.5 mg weekly) and folic acid (5.0 mg weekly), along with paracetamol for pain, to reduce the side effects of NSAIDs. On follow‐up, her pain decreased gradually, her appetite improved, and she gained the ability to walk with an assistant. The ESR value decreased to 40 mm/h without any change in the liver function tests. The treatment was continued, and the patient was put on a regular follow‐up schedule. In the patient's last visit, which was 3 months after the initial presentation, the patient's condition had improved significantly, her inflammatory markers had returned to normal, and she was able to walk without an assistant.

## 3 DISCUSSION

RA is a chronic, multisystemic inflammatory disease marked by damaging synovitis. It may affect any joint; however, it is most often associated with erosive alterations in the tiny joints of the hand and foot. RA is a progressive condition that causes diminished functional ability and quality of life, as well as higher mortality and morbidity.^7^ EORA is often described as the onset of symptoms after the age of 65. Other sources describe EORA as beginning after the age of 60. The prevalence rate of EORA is close to 2%.^8^ EORA was considered a distinct clinical entity with demographic, clinical, and immunogenic features different from young‐onset rheumatoid arthritis, which occurs before the age of 65. In this regard, EORA is distinguished by a less recognizable female predominance, although our case was a female.^9^ EORA is a heterogeneous condition with three different clinical presentations. The most prevalent clinical type (70%) has rheumatoid factor positivity, joint erosions, and a worse prognosis than young‐onset rheumatoid arthritis. The second variant (25%) is a polymyalgia rheumatica (PMR)‐like form with involvement of the proximal limb joints. It is often RF‐negative, has a rapid onset, does not lead to joint erosions, and has a favorable prognosis. Asymmetric nonerosive polyarthritis may develop in 25% of PMR patients; hence, a differential diagnosis is important to be excluded. The third EORA pattern is characterized by clinical and prognostic similarity to the RS3PE syndrome (remitting seronegative symmetrical synovitis with pitting edema).^10^, ^11^ Previous studies have failed to provide a clear picture of the prognosis for EORA patients. Some studies found a better prognosis for EORA than young‐onset rheumatoid arthritis, while others found the opposite. Counseling a patient with EORA is challenging without knowing the patient's prognosis. This data will be beneficial for physicians as they consider the pros and cons of various EORA treatments. The risk of toxicity from disease‐modifying anti‐rheumatic “DMARD” medicines is higher in patients with EORA due to their advanced age, co‐occurring comorbidities, and fragility.^12^, ^13^ In EORA, the average age at symptom onset is nearly 70 years, and approximately half of patients are consulted during the first year of disease onset^9^; however, in our case, the onset was too late at the age of 106 years for a female, and as we presented her as the first case to be documented in the medical literature, the decision of treatment was difficult due to the presence of the comorbidities, despite that her condition showed significant improvement using DMARDs.

## 4 CONCLUSION

Our case highlights a rare occurrence of newly diagnosed rheumatoid arthritis in an extremely old female (106 years old) that is rarely reported in the medical literature. By reporting this case, we hope to encourage medical professionals to concentrate on diagnosing old patients with unusual presentation of rheumatoid arthritis.

## AUTHOR CONTRIBUTIONS

**Ali ALQazzaz:** Conceptualization; investigation; project administration; supervision. **Faiq I. Gorial:** Data curation; formal analysis; resources. **Ahmed Dheyaa Al‐Obaidi:** Data curation; supervision; writing – original draft; writing – review and editing. **Mustafa Najah Al‐Obaidi:** Data curation; writing – original draft; writing – review and editing. **Nabaa Ihsan Awadh:** Investigation; resources; validation; visualization; writing – review and editing. **Hashim Talib Hashim:** Project administration; writing – original draft; writing – review and editing.

## FUNDING INFORMATION

No source of funding received.

## CONFLICT OF INTEREST STATEMENT

We declare that we have no conflict of interest.

## CONSENT

Written informed consent was obtained from the patient to publish this report in accordance with the journal's patient consent policy.

## Data Availability

None.
